# Optimizing Control Definitions in Opioid Use Disorder Genetic Research Using Electronic Health Records

**DOI:** 10.1111/adb.70094

**Published:** 2026-01-19

**Authors:** Maria Niarchou, Ellen L. Tsai, Mariela V. Jennings, Annika Faucon, Hyunjoon Lee, Kritika Singh, Justin D. Tubbs, Panos Roussos, Tian Ge, Richard C. Crist, Rachel Vickers‐Smith, Howard Edenberg, Amy Moore, Bradley T. Webb, Eric O. Johnson, PsycheMERGE Substance Use Disorder Workgroup, Rachel L. Kember, Jordan W. Smoller, Lea K. Davis, Brandon J. Coombes, Georgios Voloudakis, David Burstein, Travis T. Mallard, Vanessa Troiani, Sandra Sanchez‐Roige

**Affiliations:** ^1^ Department of Medicine, Division of Genetic Medicine Vanderbilt University Nashville Tennessee USA; ^2^ Department of Genetic and Genomic Sciences Icahn School of Medicine at Mount Sinai New York New York USA; ^3^ Department of Psychiatry University of California San Diego La Jolla California USA; ^4^ Department of Biomedical Informatics Vanderbilt University Medical Center Nashville Tennessee USA; ^5^ Psychiatric and Neurodevelopmental Genetics Unit, Center for Genomic Medicine Massachusetts General Hospital Boston Massachusetts USA; ^6^ Center for Precision Psychiatry, Department of Psychiatry Massachusetts General Hospital Boston Massachusetts USA; ^7^ Department of Psychiatry Harvard Medical School Boston Massachusetts USA; ^8^ Stanley Center for Psychiatric Research Broad Institute of MIT and Harvard Cambridge Massachusetts USA; ^9^ Center for Disease Neurogenomics Icahn School of Medicine at Mount Sinai New York New York USA; ^10^ Department of Psychiatry Icahn School of Medicine at Mount Sinai New York New York USA; ^11^ Friedman Brain Institute Icahn School of Medicine at Mount Sinai New York New York USA; ^12^ Center for Precision Medicine and Translational Therapeutics JJ Peters VA Medical Center Bronx New York USA; ^13^ Mental Illness Research Education and Clinical Center JJ Peters VA Medical Center Bronx New York USA; ^14^ Department of Psychiatry Perelman School of Medicine, University of Pennsylvania Philadelphia Pennsylvania USA; ^15^ Mental Illness Research, Education and Clinical Center Veterans Integrated Service Network 4, Crescenz Veterans Affairs Medical Center Philadelphia Pennsylvania USA; ^16^ Department of Epidemiology and Environmental Health University of Kentucky College of Public Health Lexington Kentucky USA; ^17^ Department of Biochemistry and Molecular Biology Indiana University School of Medicine Indianapolis Indiana USA; ^18^ Department of Medical and Molecular Genetics Indiana University School of Medicine Indianapolis USA; ^19^ RTI International Research Triangle Park North Carolina USA; ^20^ Virginia Institute for Psychiatric and Behavior Genetics, Department of Psychiatry Virginia Commonwealth University Richmond Virginia USA; ^21^ Genomics and Translational Research Center RTI International, Research Triangle Park North Carolina USA; ^22^ Department of Psychiatry and Behavioral Sciences Vanderbilt University Medical Center Nashville Tennessee USA; ^23^ Department of Artificial Intelligence and Human Health Icahn School of Medicine at Mount Sinai New York New York USA; ^24^ Charles Bronfman Institute for Personalized Medicine Icahn School of Medicine at Mount Sinai New York New York USA; ^25^ Department of Quantitative Health Sciences Mayo Clinic Rochester Minnesota USA; ^26^ Geisinger College of Health Sciences Scranton Pennsylvania USA; ^27^ Institute for Genomic Medicine University of California San Diego La Jolla California USA

## Abstract

Amidst the opioid crisis, understanding the genetic basis of opioid use disorder (OUD) is crucial for identifying biological mechanisms and intervention points. However, genome‐wide association studies (GWASs) have been hampered by inadequate sample sizes and often the use of control populations not assessed for prior opioid exposure. Because opioid exposure is a prerequisite for the development of OUD, consideration of exposure history in controls is important. Electronic health record data (EHR) paired with genomic information allow a broader sampling of patients with OUD and exposed controls. We leveraged data across two healthcare systems to evaluate the impact of using controls not screened for opioid exposure (‘generic’) versus minimally opioid‐exposed control (‘exposed’). First, at the phenotypic level, we conducted phenome‐wide association studies (PheWAS) to compare the medical comorbidity profiles of OUD cases when using generic versus exposed controls. While PheWAS results for OUD‐related comorbidities were more pronounced when using the generic group, 83% of the disease associations were overlapping and of similar effect sizes. Second, at the genetic level, we conducted GWAS (cases vs. generic; cases vs. exposed) and assessed differences in genetic correlations and degrees of phenotypic misclassification. Genetic results were concordant across control groups based on heritability (generic: 0.16 ± 0.07 vs. 0.10 ± 0.07), associations with the coding *OPRM1* variant rs1799971 (*p*_*generic*_ = 8.83E‐03 vs. *p*_*exposed*_ = 1.83E‐02) and genetic correlations with prior OUD GWAS (*r*_*g‐generic*_ = 0.83 ± 0.26 vs. *r*_*g‐exposed*_ = 0.78 ± 0.27). Although GWASs were limited by sample size (*N*_generic_ = 6269, *N*_exposed_ = 6365), compared to an independent OUD GWAS (*N* = 425 944), the dilution value for the two GWAS was not different from 1, suggesting no major impact of phenotypic misclassification. This study represents the first effort to enhance OUD genetic research through optimization of control definitions using EHR data. Generic controls ascertained within the US health systems, where exposure to prescription opioids is high, offer a practical alternative for genetic studies of OUD.

## Graphical abstract

This study contributes to the broader discussion of control selection in opioid use disorder (OUD) research. By comparing unscreened (“generic”) versus minimally opioid‐exposed controls in phenome‐wide association analyses, generic controls produced larger effect sizes and more associations, though most results overlapped. Genetic findings were also concordant. While the selection of control groups has trade‐offs, generic controls ascertained within US health systems, where exposure to prescription opioids is high, offer a practical alternative to screened controls for OUD research.

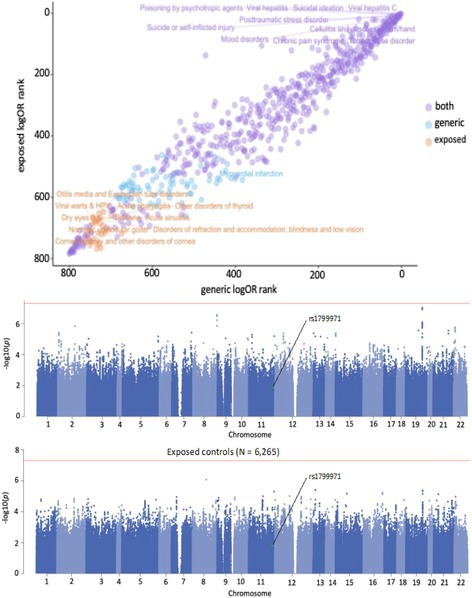

## 1 Introduction

The opioid epidemic is one of the most pressing global health challenges. In the United States, opioid overdose deaths have soared from 137 per day in 2019 to 255 per day in 2021 [1], the highest rate of fatal opioid overdoses ever reported. While recent data indicate a small decline in overdose deaths in some states [2], the opioid crisis remains a significant public health challenge [3], accounting for more accidental deaths than motor vehicle accidents, falls or firearms [2, 4]. In the United States, over two million people are suffering with an opioid use disorder (**OUD** [4, 5]), and the yearly economic burden is estimated to be approximately $140 billion [6]. Given the high rate of relapse associated with OUD, the fallout from the current epidemic will persist for decades [7].

Both genetic and environmental factors influence the risk for OUD [8, 9, 10]. Genome‐wide association studies (GWASs) can identify loci that confer risk for OUD and can provide a window into the biological mechanisms involved in opioid addiction. Although the effect sizes of individual risk alleles are small, their combined influence, captured through polygenic scores (PGS), can confer substantial risk [11]. PGS can improve risk prediction and disease stratification and enable precision medicine approaches. However, existing OUD GWAS efforts continue to be hampered by relatively small sample sizes and frequent selection of more extreme cases. Such enrichment can improve power to detect associations but may bias effect size estimates and limit the applicability of findings to individuals with less severe or subthreshold OUD, thereby reducing generalizability to broader populations.

The growing availability of electronic health records (EHR) with associated genomic data in large biobank cohorts offers new opportunities to advance the discovery of additional loci associated with OUD [12, 13, 14]. For example, OUD can be characterized using billing codes based on the International Classification of Diseases (ICD), thereby amassing very large numbers of individuals in a cost‐effective manner. While ICD codes are not designed for research purposes and lack the depth of clinical interviews conducted by trained clinicians [15, 16, 17, 18, 19], they still provide a means of identifying OUD in real‐world healthcare settings. Using this approach, our group and others have identified over 14 genomic loci associated with problematic opioid use and OUD [9, 20, 21, 22, 23, 24, 25, 26, 27].

Another potential limitation of many OUD GWAS is that control populations have not systematically been assessed for prior opioid exposure. Because exposure to the drug is a prerequisite for the development of OUD, consideration of drug exposure history in controls may be particularly important. Failing to account for differences in exposure can cause causal inference challenges, such as limiting our ability to distinguish between genetic liability for OUD and genetic liability for opioid exposure itself. In theory, including controls with documented opioid exposure could enhance statistical power by reducing heterogeneity (defined here as differences in opioid exposure history and related factors) between cases and controls, thereby allowing for a more accurate assessment of genetic liability for the progression from opioid use to OUD [27]. Such an approach could provide a more comparable reference group by accounting for shared environmental and behavioural factors that influence exposure risk, potentially uncovering genetic variants specifically associated with the disorder rather than general opioid use. Alternatively, only including controls with documented opioid exposure could inadvertently select for individuals with genetic factors that increase opioid exposure but decrease risk for OUD (e.g., genetic variants associated with pain sensitivity but not addiction vulnerability). In contrast, the use of controls unscreened for opioid exposure may introduce greater heterogeneity, potentially reducing statistical power. For instance, including controls who were never prescribed opioids could inadvertently select for individuals with lower genetic risk for chronic pain, which could confound OUD genetic associations. However, including generic controls is easier to implement, often allows for larger sample sizes and may be less problematic in settings where opioid exposure is widespread, such as hospital populations.

Despite these potential advantages, the impact of using controls with differing opioid exposure characterization on genetic association results remains largely untested [24]. Furthermore, there is currently no consensus on how to define exposure across studies [28], ranging from short‐term to chronic prescription use [28].

Leveraging data from two healthcare systems, we assessed the impact of using phenotypic and genetic data from individuals with minimal exposure to prescription opioids (‘exposed controls’, i.e., at least two opioid prescriptions no more than 90 days apart and no third one within 9 months of the second prescription to avoid including patients with chronic opioid use [29]), compared to a more traditional control definition that merely requires the controls not to have the case diagnosis (‘generic controls’). First, we examined how the comorbidity profile associated with an OUD diagnosis differs depending on the choice of control group (exposed vs. generic) by conducting phenome‐wide association studies (PheWAS) across thousands of medical conditions. We hypothesized that effect sizes would be smaller when using exposed controls because these individuals are more likely to share clinical and behavioural characteristics with OUD cases (e.g., pain conditions requiring opioid therapy, psychiatric comorbidities or other substance use disorders) based on our prior work [28], thereby reducing the contrast in risk between groups. Second, we evaluated how potential levels of heterogeneity introduced by the choice of control group affect the genetic associations with OUD by conducting GWAS, estimating genetic correlations with prior large‐scale GWAS of OUD and related traits [20, 25, 29, 30, 31, 32, 33, 34, 35], and quantifying the level of phenotypic misclassification using the Phenotypic Measurement of Effective Dilution (PheMED) software [36]. We hypothesized that using exposed controls would reduce heterogeneity in opioid exposure among controls and thereby improve the power to detect genetic associations with OUD compared to using generic controls.

## 2 Subjects and Methods

### 2.1 Study Cohorts

Two health systems were included in our analyses: Vanderbilt University Medical Center (VUMC) and Mass General Brigham (MGB). VUMC is a tertiary care center that provides inpatient and outpatient care in Nashville, Tennessee. The VUMC EHR was established in 1998 and contains the medical records of 3.2 million patients, spanning more than 20 years of longitudinal data. The synthetic derivative (SD) database is a de‐identified mirror image of the VUMC EHR, including data from various sources such as billing codes from the International Classification of Diseases, 9th and 10th editions (ICD‐9 and ICD‐10) and prescription medications. In 2007, VUMC launched BioVU, a biobank linked to the SD database that stores DNA from over 310 000 patients. The study was reviewed and approved by the VUMC institutional review board (IRB #160302, #172020 and #190418).

The MGB Research Patient Data Registry (https://rpdrssl.partners.org/) is an EHR database that spans more than 20 years of data from over 6.5 million patients seen in Massachusetts General Hospital, Brigham and Women's Hospital, and other community and specialty hospitals in the Boston area. The database includes data on ICD‐9 and ICD‐10 codes and prescription medications and is linked to the MGB biobank (MGBB), with DNA from over 60 000 patients. The study was reviewed and approved by the MGB institutional review board (IRB approval #2018P002642).

### 2.2 Cohort Definitions

We defined cases based on having at least one OUD ICD code (see Table S1 for the list of codes), consistent with prior work [20].

**FIGURE 1:**
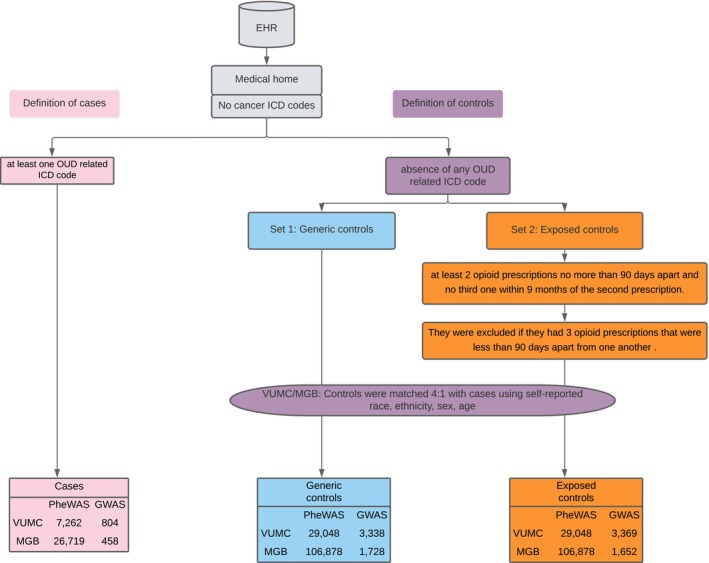
Study design flowchart. We limited our data to patients for whom Vanderbilt University Medical Center (VUMC) and Mass General Brigham (MGB) were likely the primary sources of care based on a minimum data floor (‘medical home’) definition. We applied a minimum data floor of five ICD codes on separate days over a 3‐year period to all individuals, regardless of case or control status, to ensure adequate longitudinal data capture and reduce the likelihood of missing relevant diagnoses due to sporadic or incomplete records. Patients with a cancer diagnosis were excluded due to the likelihood for long‐term analgesia for cancer‐related pain. Cases were identified as those with any OUD ICD code (see Table S1 for list of codes). We selected two control groups: (a) ‘generic controls’ not screened for prior prescription opioid use and (b) ‘exposed controls’ (i.e., at least two opioid prescriptions no more than 90 days apart and no third one within 9 months of the second prescription). Exposed controls were also required to not have 3 opioid prescriptions that were less than 90 days apart from one another to avoid chronic use. Both control groups excluded individuals with any OUD ICD code. All control patients were also required to be at least 18 years old to reduce the likelihood of including individuals who had not yet developed OUD. Controls were matched 4:1 with cases for both phenome‐wide association analyses (PheWAS) and genome‐wide association studies (GWASs).

We selected two nonoverlapping control groups of equivalent sample sizes (see Figure 1 for a flowchart of our analyses). Both groups excluded individuals with any OUD ICD code but differed in the screening for prior prescription opioid use: (a) controls not screened for prior prescription opioid use (‘generic’ control group that included people who may have or may not have been exposed to opioid prescriptions) and (b) minimally prescription opioid‐exposed controls (‘exposed’). Patients in the exposed group received at least two opioid prescriptions no more than 90 days apart and no third one within 9 months of the second prescription. Exposed controls were also required to not have three opioid prescriptions that were less than 90 days apart from one another. This definition of exposed control was intended to capture individuals with meaningful but limited opioid exposure enough to confirm access and use while excluding those with patterns consistent with long‐term or chronic opioid therapy, who are at higher risk of undiagnosed OUD. This approach reduces heterogeneity in the exposed control group by avoiding inclusion of individuals with either negligible exposure (single prescription) or prolonged high‐dose use, both of which could confound associations. These definitions, which are specific to EHR‐linked prescription data and may not generalize to non‐EHR cohorts, have been described in our prior work [28].

We limited our data to patients for whom VUMC or MGBB was likely the primary source of care based on a minimum data floor definition that required the presence of at least five ICD codes for any medical condition on different days over a 3‐year period to all individuals regardless of case control status. This helps to reduce the bias that could arise from systematic missing values in the data. Control patients were also required to be at least 18 years old at the time of the analysis to reduce the likelihood of including individuals who had not yet developed OUD. Finally, we also excluded patients with a cancer diagnosis from both cases and controls (see Table S2 for cancer‐related ICD codes) due to the likelihood of long‐term analgesics being prescribed for cancer‐related pain.

### 2.3 Phenome‐Wide Association Analyses (PheWAS)

To investigate OUD disease patterns across control groups, we conducted two PheWAS at the VUMC and MGB sites: one comparing medical comorbidities between OUD cases and generic controls and the other between OUD cases and exposed controls (predictor variable: case/control; outcome variable: medical phenotype). The phenotypes tested in the PheWAS were derived by mapping ICD‐9 and ICD‐10 codes to 1817 ‘phecodes’, as described and validated by the Phecode Map 1.2b [37]. Phecodes use a standardized vocabulary and can denote diseases, traits, or symptoms (http://phewascatalog.org). For each phecode analysed, we required a minimum of 100 cases. Controls were matched in a 4:1 ratio with cases based on EHR‐reported race, ethnicity, sex and median age of record using the function *matchit* in R. The results from both sites were then meta‐analysed using fixed effects in METAL [38]. To assess heterogeneity, we calculated *I* ^*2*^ statistics in METAL to identify associations that differed in effect size between health systems. Phecodes with *I* ^*2*^ > 0.75 were considered significantly heterogeneous [39].

As a supplementary analysis, we also examined potential differences in the disease profiles between generic and exposed controls and meta‐analysed that across sites (see Note S1 and Table S5).

Associations between OUD and the two control groups were compared by fitting logistic regression models with the PheWAS v0.12 R package [37]. The number of phecodes tested varied by analysis (see Tables S3–S5), and a Bonferroni correction was applied to account for multiple testing to adjust the significance threshold for each PheWAS.

### 2.4 GWAS

To compare the impact of control definitions on genome‐wide associations, we performed two OUD GWASs (cases vs. generic; cases vs. exposed) within each health system. Genotyping used the Illumina MEGA_ex platform (VUMC, MGBB) and Global Screening Array (MGBB), with imputation at both sites via the Michigan Imputation Server [40] and the Haplotype Reference Consortium reference panel [41]. Single nucleotide polymorphisms (SNPs) with imputation quality (*R* ^2^ > 0.3 in VUMC; *R* ^2^ > 0.6 in MGBB) were converted to hard genotype calls. Variants were retained only if they passed the imputation quality threshold at both sites, ensuring comparability across datasets. We applied an IBD filter of 0.2 to exclude cryptic relatedness and restricted the analyses to individuals most genetically similar to European reference populations based on principal components (PCs); this choice was made due to the limited number of cases (*N* < 201) in individuals genetically similar to other non‐European reference populations [42]. We also applied a minor allele frequency (MAF) filter of 0.05. Details on genotyping and quality control are available for VUMC [43] and MGBB [35].

Univariate GWASs were conducted at each site using PLINK 2.0 [44], adjusting for age, sex, age×sex interaction, age^2^ and the first 20 PCs. GWASs across sites were meta‐analysed (meta‐GWAS) using METAL [38] with the standard error analysis scheme.

The functional variant rs1799971 in *OPRM1* (μ‐opioid receptor gene, the main biological target for opioid drugs) has been significantly associated with OUD in prior GWASs [20, 24]. In a hypothesis‐driven analysis, we compared summary statistics for rs1799971 across the two meta‐GWASs.

### 2.5 Linkage Disequilibrium Score Regression (LDSC)

We estimated SNP‐based heritability (*h* ^2^_*SNP*_) and genetic correlation (*r*_*g*_) using LDSC [45]. Meta‐GWASs were munged to HapMap3 SNPs. We used LDSC to calculate genetic correlation with prior GWAS of OUD and other OUD‐related traits (Table S6) [20, 25, 29, 31, 32, 33, 34, 35]. These traits were selected because they were well powered (*N* > 100 000) and exhibited a strong genetic correlation with OUD in previous studies. The standard Benjamini–Hochberg false discovery rate correction (FDR 5%) was applied for multiple testing.

### 2.6 PheMED

To quantify the level of phenotypic misclassification across GWAS, we used the PheMED software [36]. The misclassification level is represented by the dilution value. PheMED leverages summary statistics from GWAS to estimate the extent of phenotypic misclassification bias, which occurs when individuals classified as cases or controls do not accurately represent their true disease status. The dilution value produced by PheMED reflects the relative SNP‐disease association effect size dilution between the studies. Higher dilution values suggest a higher degree of bias, meaning that the phenotypic definitions used in the GWAS may have introduced noise into the genetic signal. Lower dilution values suggest better classification and more reliable genetic associations. Using an independent large‐scale OUD GWAS as a reference point [20] (dilution value of 1), we computed the dilution value and 95% confidence intervals (CIs) for the two meta‐GWASs (OUD vs. generic controls; OUD vs. exposed controls). Comparing these values allowed us to evaluate the relative accuracy of the phenotypic definitions used across the meta‐GWAS.

## 3 Results

### 3.1 PheWAS

**FIGURE 2:**
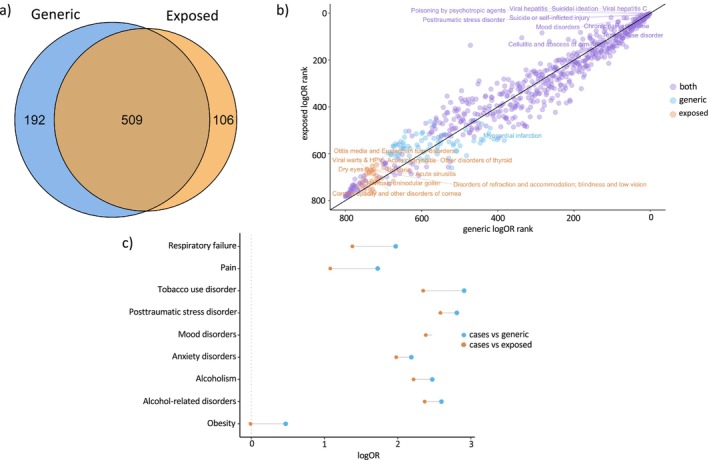
Phenome‐wide association meta‐analyses of opioid use disorder using two control populations (generic vs. minimally opioid‐exposed). (a) Across both control groups, OUD case status was associated with > 600 medical conditions, 509 of them overlapping. (b) While the associations were mostly consistent (purple), there was also specificity (orange, blue). (c) The associations are of higher magnitude in the generic group.

The total sample of the meta‐PheWAS included 33 981 cases compared to 135 926 generic and 135 926 exposed matched controls (Figure 1). A large proportion (82.73%) of individuals in the generic group had at least one mention of opioid prescription medication in their records. PheWAS identified 701 statistically significant associations between OUD cases and generic controls (Table S3) and 615 statistically significant associations between OUD cases and exposed controls (Table S4). Of these, 509 were common between the two analyses (Figure 2A), 192 were unique to the generic group, and 106 were unique to the exposed group (Figure 2B); counts reflect associations that reached statistical significance within each PheWAS. Moreover, none of the associations unique to the generic or the exposed group were enriched in a particular phecode group.

The generic group consistently showed greater effect sizes compared to the exposed group (Tables S3 and S4). As hypothesized, this was particularly true for traits comorbid with OUD (Figure 2C). For example, the odds ratio (OR and 95% CI) for tobacco use disorder was 18.33 [18.28–18.35] in generic controls versus 10.47 [10.44–10.50] in exposed controls. Similarly, ORs for posttraumatic stress disorder were larger in generic controls (16.56 [16.52–16.63] vs. 13.25 [13.20–13.30]), and so was the OR for alcohol use disorder (AUD) (11.86 [11.81–11.90] vs. 9.18 [9.14–9.23]), major depressive disorder (9.41 [9.38–9.45] vs. 7.33 [7.30–7.36]), among others (Figure 2C).

While we observed that effect size estimates were statistically different across 595 traits (95.2%) between the two control groups (Figure 2B), the directions of effect were largely similar (Tables S3 and S4).

We examined potential differences in the disease profiles between generic and exposed controls. Of the 461 significant associations identified in both generic and exposed controls (Table S5), 99% were in the direction of exposed controls being at higher risk compared to generic controls, including associations with pain conditions (e.g., chronic pain, 1.42 [1.40–1.45]) and psychiatric disorders (e.g., AUD, 1.34 [1.29–1.38]; major depressive disorder, 1.26 [1.23–1.28]; Supporting Information).

### 3.2 GWAS and SNP‐Heritability

**FIGURE 3:**
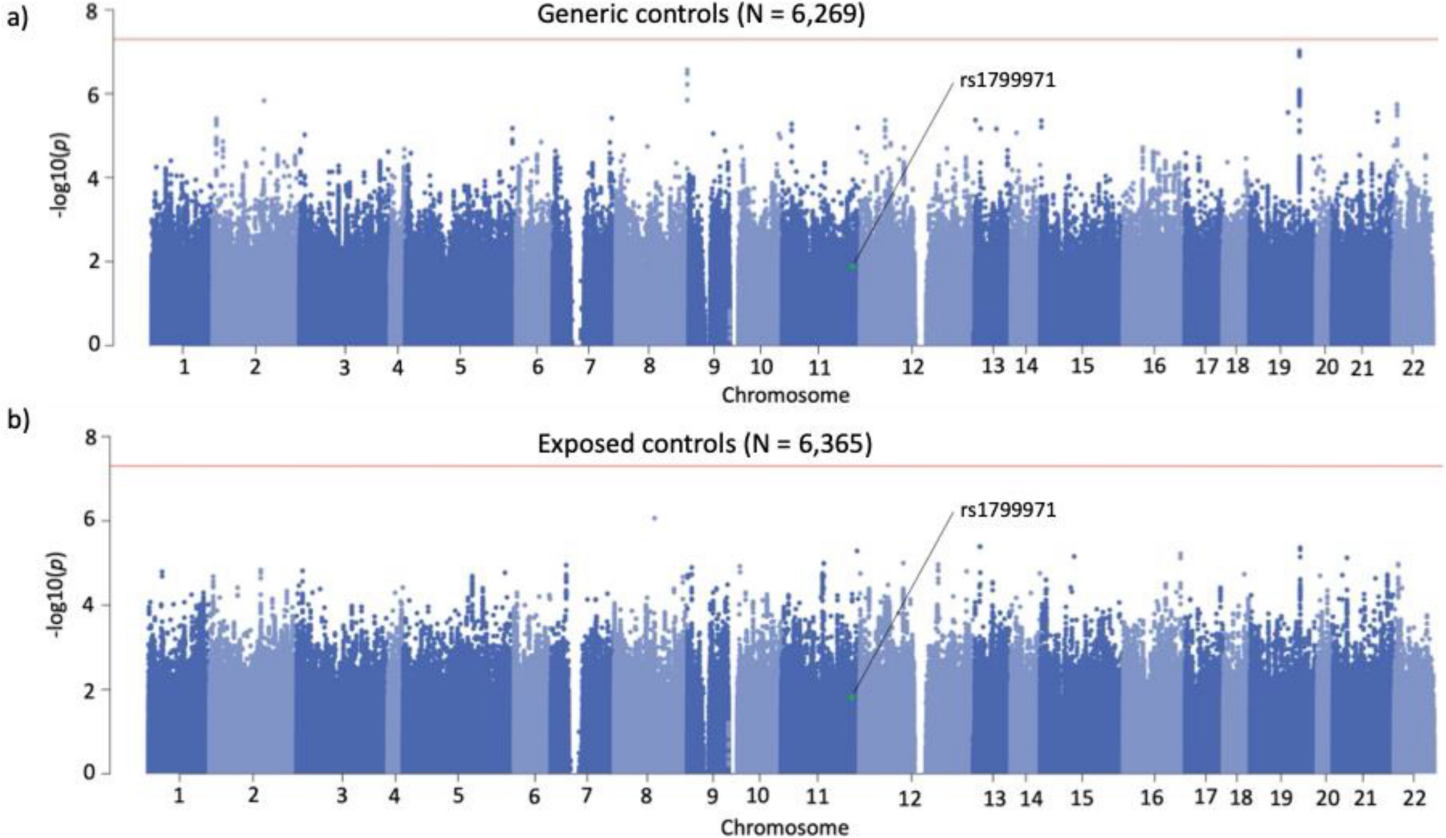
Genome‐wide association analyses of OUD using generic (a) and minimally opioid‐exposed controls (b). Associations with coding *OPRM1* SNP rs1799971 (green dot) are comparable across control groups (*p* = 1.50E‐02 vs. *p* = 1.30E‐02). Lambda Gc_generic_ = 1.019 Mean *χ* ^2^: 1.019, Intercept: 0.998 (0.006) vs. Lambda Gc_exposed_ = 1.014 Mean *χ* ^2^: 1.012, Intercept: 0.998 (0.006).

There were no genome‐wide significant (*p* < 5.00E‐08) associations in either of the meta‐GWAS (*N*_generic_ = 6269, *N*_exposed_ = 6365; Figure 3). Associations with coding *OPRM1* SNP rs1799971 were similar across the generic (*p* = 1.31E‐02; β = 0.17) and exposed (*p* = 1.52E‐02; β = 0.17) groups. The *h* ^*2*^_*SNP*_ was significant in both groups (*h* ^*2*^_*SNP*‐generic_ = 0.16 ± 0.07 vs. *h* ^*2*^_*SNP*‐exposed_ = 0.10 ± 0.07), but *h* ^*2*^_*SNP*_ estimates were not statistically different (*p* = 0.53) across the groups.

### 3.3 Genetic Correlations

Inspection of standard error estimates showed that the genetic correlations with OUD‐related traits were similar across the generic and exposed groups, including OUD (*r*_*g‐generic*_ = 0.83 ± 0.26 vs. *r*_*g‐exposed*_ = 0.78 ± 0.27) and general addiction (*r*_*g‐generic*_ = 0.99 ± 0.45 vs. *r*_*g‐exposed*_ = 0.85 ± 0.40), among others (Table 1; Table S6).

**TABLE 1:** Heritability and genetic correlation (*r*_*g*_) estimates were not statistically different across groups.

	Generic controls		Exposed controls	
Heritability	0.16 ± 0.07		0.10 ± 0.07	
Genetic correlation	*rg*	*P*	*rg*	*P*
OUD	0.83 ± 0.26	1.42E‐03	0.78 ± 0.27	3.80E‐03
Problematic opioid use	0.85 ± 0.30	4.86E‐03	0.75 ± 0.35	3.49E‐02
Addiction factor	0.99 ± 0.45	2.59E‐02	0.85 ± 0.40	3.58E‐02
Externalizing	0.58 ± 0.16	3.39E‐04	0.66 ± 0.25	6.80E‐03
Tobacco use disorder	0.73 ± 0.21	6.20E‐04	0.56 ± 0.18	2.20E‐03
Problematic alcohol use	0.58 ± 0.14	4.08E‐05	0.65 ± 0.23	5.60E‐03
Multisite chronic pain	0.58 ± 0.16	2.09E‐04	0.59 ± 0.23	1.08E‐02
Depression	0.48 ± 0.12	9.14E‐05	0.54 ± 0.21	8.30E‐03
PTSD	0.58 ± 0.14	4.08E‐05	0.65 ± 0.23	5.60E‐03

### 3.4 PheMED

Compared to the latest OUD GWAS [20], the dilution value for the two meta‐GWASs was not different from 1 (for generic controls: PheMED value (95% CI) = 0.83 [0.66–1.10], *p* = 0.16; for exposed controls: PheMED value (95% CI) = 1.04 [0.80–1.49], *p* = 0.80), suggesting minimal impact of phenotypic misclassification on the results.

## 4 Discussion

This study is the first to evaluate how different levels of opioid exposure, as documented by opioid prescription histories, affect OUD GWAS within EHR settings as well as causal inferences that can be drawn from the results. Leveraging data from two healthcare systems, we first assessed the phenotypic heterogeneity of two control definitions (generic vs. minimally exposed). PheWAS showed stronger associations in both the magnitude of effect sizes and the number of associations with medical conditions when comparing individuals with OUD diagnosis to generic controls rather than exposed controls. In other words, on average, the mean trait difference between cases and generic controls was larger than between cases and minimally exposed controls. However, most of the PheWAS associations were overlapping, suggesting that the two control groups were more similar than expected. For some psychiatric and substance use phenotypes (e.g., suicidal ideation), we observed large ORs (> 10) in both groups, consistent with the high comorbidity of these conditions with OUD, as we have previously shown [46]. Next, we conducted a series of genetic analyses to evaluate whether and how the choice of control group affects the genetic associations with OUD. Although genetic analyses were limited by sample size, GWAS findings, genetic correlations with OUD and other related traits, as well as assessment of potential phenotypic misclassification, did not reveal substantial differences across the two control groups. Further examination showed that over 80% of individuals in the generic group had a history of opioid prescription in their records. These findings suggest that including generic controls not assessed for prior opioid prescription use, at least ascertained in medical settings, may not introduce high levels of phenotypic heterogeneity compared to using minimally exposed controls, thereby serving as a more effective strategy to increase sample sizes and improve gene discovery for OUD.

In theory, minimally exposed controls should be better matched to cases with regard to nonspecific OUD comorbidities, such as acute pain or risk‐taking [27]. While we indeed observed fewer associations in the comorbidity profile when we used exposed controls compared to generic controls, we also found a large overlap in the PheWAS associations across the two groups. This similarity could be explained by the fact that a large proportion of the generic controls (82.73%) in our sample had a history of opioid prescription. Such a large prevalence can be attributed to the way that this sample was ascertained—tertiary hospitals within two US states where prescription opioid practices are prevalent and a wide range of medical conditions is more common than in the general population. These findings may not generalize to settings where opioid prescription use is not as prevalent, such as in population‐based cohorts or populations outside of the United States. However, our results are consistent with the findings of Gaddis et al. [24], which observed similarly high genetic correlations between opioid addiction cohorts using exposed and unscreened controls ascertained outside the medical system. Pending further studies that provide empirical evidence to the contrary, maximizing sample size by inclusion of generic controls may optimize opportunities for variant association discoveries for OUD. However, we note that if the level of exposure is low, results may be more relevant to exposure to prescription opioids than to risk for OUD. Therefore, we encourage that future studies systematically characterize the level of opioid exposure in their population, whenever possible, prior to study design.

Similarly, we also originally hypothesized that using exposed controls would reduce heterogeneity (i.e., fewer differences in opioid exposure history and related factors) and improve the power to detect genetic associations with OUD compared to using generic controls. However, we observed no significant differences in GWAS findings or heritability estimates between the two control populations; however, the lack of power could affect heritability estimates and would likely benefit from larger sample sizes. Similarly, genetic correlation estimates between our OUD GWAS and prior large‐scale OUD GWAS [20], as well as other opioid‐related GWAS, were similar across the two control populations. Furthermore, there was no substantial evidence of phenotypic misclassification across control groups when compared to the largest current OUD GWAS [20]. While the definition of cases in the previous OUD GWAS was identical to ours, all controls in that study were exposed to prescription opioids. On the other hand, in our study, ~20% of the generic controls had no evidence of opioid prescription in their records. These series of results suggest that genetic signals, which have small effect sizes [9, 10], remain stable regardless of the discrepancies. Prior examination of unscreened and population controls has shown similar consistency of genetic signal compared to exposed controls [24]. Further research could establish a threshold at which exposure levels may lead to biassed genetic associations.

The topic of control group selection has been previously discussed in the field of psychiatric genetics [47, 48, 49, 50]. These findings align with prior recommendations to use generic controls for opioid exposure, particularly when the level of exposure in the control population is expected to be high, as is the case for studies of PTSD [51, 52] in populations where exposure to traumatic experiences is also high. Although in theory, exposed controls may be a more optimal control population, their inclusion is challenging. First, there is no consensus on how to best measure opioid exposure, ranging from a single prescription to long‐term, high‐dose use. Second, this variability in opioid exposure risks misclassification, as some exposed controls, particularly those with long‐term exposure, may have undiagnosed OUD or opioid‐related conditions without meeting diagnostic criteria. Similarly, the criteria used to define opioid exposure can introduce bias, influencing the composition of the comparison group. For example, requiring multiple opioid prescriptions to define exposure may select for individuals with more severe pain, potentially biasing comparisons with the OUD group by conflating pain severity with opioid use. Third, nonmedical or illicit use of opioids is rarely documented in the medical records. Lastly, ascertaining opioid exposure (which is not always available) inevitably results in lower sample sizes than otherwise available, which may limit our ability to identify risk variants for OUD, particularly in non‐European genetic ancestry populations. Researchers should weigh trade‐offs between control group differences and data availability for OUD genetic studies.

Our study is not without limitations. To define cases, we required an instance of at least one OUD ICD code and showed this effectively captures OUD‐related comorbidities, consistent with a prior study [20]. However, this method may miss cases due to incomplete diagnostic records [15, 16, 17, 18, 19]. Future studies should integrate structured (e.g., toxicology results) and unstructured data (e.g., clinical notes) to improve case ascertainment and phenotypic accuracy. Improving phenotypic classification also applies to control populations. Future studies could also examine how control selection may lead to other causal inference problems that may affect GWAS results (e.g., gene–environment interaction, generalizability issues; Note S1). We expect that incorporating additional variables, such as survey responses in cohorts like *All of Us* [53], will allow us to further refine control classification. Finally, although gene discovery was not the focus of our study, the GWASs were limited by sample size, reducing power for downstream analyses, including heritability estimation, genetic correlation, and misclassification assessment. While these findings require replication in larger samples, the strong genetic correlations with other OUD GWAS support their inclusion in future larger studies to improve power for detecting novel associations.

## 5 Conclusions

Our findings contribute new insights to the broader discussion of control selection in OUD GWAS. While the selection of control groups has trade‐offs, generic controls ascertained within the US health systems, where exposure to prescription opioids is high, offer a practical alternative to screened controls for genetic studies of OUD.

## Author Contributions

S.S.‐R. and M.N. conceived and designed the study. M.N., E.T., M.V.J., A.F., H.L. and T.T.M. curated the data. Formal analyses were conducted by M.N., E.T., M.V.J., A.F., H.L. and T.T.M. Methodological development was led by M.N. and S.S.‐R. S.S.‐R. and M.N. supervised the project. M.N., S.S.‐R., E.T., M.V.J., H.L. and T.T.M. drafted the manuscript, and all authors contributed to the review and editing of the final version.

## Funding

L.K.D., V.T., and S.S.R. are funded through the National Institute on Drug Abuse (NIDA DA054071). S.S.R. is also funded through the National Institute on Drug Abuse (NIDA) DP1DA054394 and 1R01DA061977‐01. The project described was supported by the National Center for Research Resources, grant UL1 RR024975‐01, and is now at the National Center for Advancing Translational Sciences, grant 2 UL1 TR000445‐06. The project was also supported by the National Institute of Mental Health (NIMH R01MH137220). J.W.S. was supported in part by 1R01MH118233. The content is solely the responsibility of the authors and does not necessarily represent the official views of the NIH. The dataset(s) used for the analyses described were obtained from Vanderbilt University Medical Center's BioVU, which is supported by numerous sources: institutional funding, private agencies and federal grants. These include the NIH funded Shared Instrumentation grant S10RR025141 and CTSA grants UL1TR002243, UL1TR000445 and UL1RR024975. Genomic data are also supported by investigator‐led projects that include U01HG004798, R01NS032830, RC2GM092618, P50GM115305, U01HG006378, U19HL065962, R01HD074711 and additional funding sources listed at https://victr.vumc.org/biovu‐funding/.

## Conflicts of Interest

Kritika Singh is now an employee at Bristol Myers Squibb and receives financial remuneration; however, all work pertaining to this paper were performed while she was a graduate student at Vanderbilt University. All other authors report no conflicts of interest.

## Supporting information


**Note S1:** Causal inference challenges that may influence OUD GWAS.

**Table S1:** OUD ICD codes.
**Table S2:** Cancer‐related ICD codes.
**Table S3:** Meta‐analysis PheWAS results between OUD cases and generic controls (Bonferroni p = 6.47E‐05).
**Table S4:** Meta‐analysis PheWAS results between OUD cases and minimally exposed controls (Bonferroni p = 6.60E‐05).
**Table S5:** Meta‐analysis PheWAS results between generic and exposed controls (Bonferroni p = 8.49E‐05).
**Table S6:** Genetic correlations between GWAS of OUD and controls (generic and minimally exposed) and select traits.

## Data Availability

Regarding the EHR samples, due to data sharing restrictions related to privacy concerns in the EHR, the datasets generated from our hospital populations will not be publicly available.
