# Supplementary material for: Optimizing Control Definitions in Opioid Use Disorder Genetic Research Using Electronic Health Records
Source: Addict Biol. 2026 Jan 19;31(1):e70094. [Article in Spanish] doi: 10.1111/adb.70094 (PMC12813555; doi:10.1111/adb.70094)
Supplement: Supplementary file 1 — Note S1: Causal inference challenges that may influence OUD GWAS. [file ADB-31-e70094-s002.docx]

**Supplementary Materials**

**Supplementary Results**

**PheWAS of generic vs minimally-exposed controls**

We examined potential differences in the comorbidity profiles between generic and minimally-exposed (“exposed”) controls and meta-analyzed across sites.

There were 461 significant associations between generic and exposed controls (**Supplementary Table 5**). Of those, 99% were in the direction of exposed controls being at higher risk compared to unscreened controls. Among the strongest associations were associations with benign neoplasm of colon, obesity, pain in joint, and back pain. The top three largest effect sizes were for other hereditary hemolytic anemias (OR=3.06[2.89-3.23], *p=*1.5x10^-37^), acute appendicitis (OR=2.99[2.92-3.08], p=3.07x10^-172^) and appendicitis (OR=2.99[2.92-3.07], p=6.10x10^-186^).

The associations that indicated higher risk for the generic control group compared to the exposed were: schizophrenia and other psychotic disorders, encounter for long-term use of antibiotics, pulmonary insufficiency or respiratory failure following trauma and surgery, multiple sclerosis, chronic pain syndrome and IIeostomy status (**Supplementary Table 5**).

**Supplementary Note 1.** Causal inference challenges that may influence OUD GWAS.

| **Causal inference challenge** | **General description** | **Specific examples** |
| --- | --- | --- |
| Confounding by Indication | Opioid exposure is often driven by medical conditions (e.g., post-surgical pain, chronic pain). If controls include individuals who were never prescribed opioids because they had fewer health conditions requiring pain management, genetic differences associated with health status (rather than OUD) may confound results. | Including controls who were never prescribed opioids could inadvertently be selecting for individuals with lower genetic risk for chronic pain, which could confound OUD genetic associations. |
| Collider Bias | If cases (OUD) and controls (non-OUD) are selected based on opioid exposure, but exposure itself is influenced by genetic and environmental factors, this could induce a spurious association between genetic variants and OUD. | Only including controls with documented opioid exposure could inadvertently be selecting for individuals with genetic factors that increase opioid exposure but decrease risk for OUD (e.g., genetic variants associated with pain sensitivity but not addiction vulnerability). This could distort GWAS associations. |
| Selection Bias Due to Varying Exposure Definitions | The way opioid exposure is defined across studies could influence the observed genetic associations. If exposure is defined as any opioid prescription in some datasets but as chronic opioid use in others, this variation could lead to biased results. | If a GWAS includes controls who had only short-term opioid prescriptions in one cohort but chronic prescriptions in another, the genetic background of controls may vary significantly, introducing inconsistencies in association results. |
| Gene-Environment Interaction Misclassification | If opioid exposure interacts with genetic predisposition to OUD, but exposure is not properly classified in controls, gene-environment interactions may be obscured. | Individuals with short- vs long-term opioid prescription use may differ in characteristics related to pain sensitivity and metabolism of opioids, affecting the interpretation of the GWAS results. |
| Generalizability Issues | If the study population is drawn from regions with differing opioid prescribing practices, differences in genetic associations may reflect prescribing patterns rather than true genetic effects. | GWAS that include cohorts from countries with strict opioid prescribing laws alongside populations with more widespread opioid access may capture differences in genetic associations driven by healthcare system effects rather than true genetic liability for OUD. Accounting for opioid exposure history in controls is essential to mitigate these biases and ensure valid causal inference. |
